# The Inhibitory Effect of Anastrozole on the Metabolism of Abemaciclib *In Vitro* and *In Vivo*

**DOI:** 10.1021/acsomega.6c00144

**Published:** 2026-03-30

**Authors:** Le-hao Jin, Zhe-yan Zhang, Xiao-yu Xu, Zhong-xi Chen, Jing Chen, Yu-bin Lan, Liu-liu Pan, Xiao-dan Zhang

**Affiliations:** a Wenzhou Seventh People’s Hospital, 26453 Wenzhou Medical University, Wenzhou, Zhejiang 325035, P.R. China; b School of Pharmaceutical Sciences, 26453 Wenzhou Medical University, Wenzhou, Zhejiang 325035, P.R. China; c Department of Obstetrics and Gynecology, The Second Affiliated Hospital and Yuying Children’s Hospital of Wenzhou Medical University, Wenzhou 325027, P.R. China

## Abstract

Anastrozole and abemaciclib are clinically recommended in combination for the treatment of advanced breast cancer. However, the potential drug–drug interaction between them remains poorly characterized, posing a potential risk for altered drug exposure and toxicity. This study aimed to systematically investigate the effect of anastrozole on the metabolism and pharmacokinetics of abemaciclib. Using a combined *in vitro* and *in vivo* approach, we characterized this interaction via UPLC-MS/MS analysis of abemaciclib and its major metabolites (M2 and M20). *In vitro* enzymatic assays demonstrated that anastrozole significantly inhibits the metabolism of abemaciclib. Consistent with these findings, *in vivo* studies in female Sprague–Dawley rats showed that 7-day pretreatment with anastrozole prior to abemaciclib administration substantially decreased abemaciclib clearance, resulting in markedly increased systemic exposure (AUC_0–t_, AUC_0–∞_, and *C*_max_). Given that abemaciclib is predominantly metabolized by CYP3A4, we further examined whether genetic polymorphisms in this enzyme influence the observed interaction. Our results revealed that CYP3A4 genetic polymorphisms significantly modulate the inhibitory potency of anastrozole. These findings indicate that anastrozole acts as a CYP3A4 inhibitor, altering abemaciclib disposition in a genotype-dependent manner. This study highlights a clinically significant drug interaction with potential toxicological implications and provides a mechanistic basis for genotype-guided therapy in patients receiving this combination regimen.

## Graphical abstract

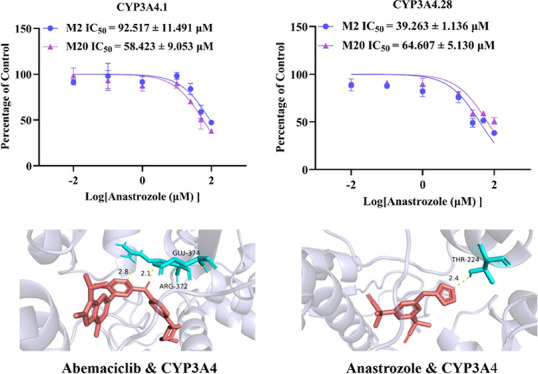

## 1 Introduction

Hormone receptor-positive (HR+) and human epidermal growth factor receptor 2-negative (HER2-) breast cancer is the most common subtype of breast cancer.^1^ In recent years, its treatment strategies have seen significant advancements with the advent of cyclin-dependent kinase 4/6 (CDK4/6) inhibitors.^2^ Abemaciclib, a highly selective and orally effective CDK4/6 inhibitor, specifically inhibits the activity of the CDK4/6-cyclin D complex, blocking the phosphorylation of the retinoblastoma protein. This action induces G1/S phase cell cycle arrest and suppresses tumor cell proliferation.^3,4^ As a third-generation targeted drug, abemaciclib has demonstrated remarkable clinical benefits in the first- or second-line treatment of HR+/HER2- advanced breast cancer. It has become an important therapeutic option for metastatic breast cancer, particularly when used in combination with endocrine therapies such as aromatase inhibitors or fulvestrant, as it significantly prolongs progression-free survival.^5,6^

However, the widespread clinical application of abemaciclib is challenged by drug–drug interactions, primarily due to its pharmacokinetic properties. Abemaciclib is well-absorbed after oral administration, with a time to peak concentration of approximately 4–6 h, and its bioavailability is only slightly affected by a high-fat diet.^7,8^ It is widely distributed in the body, with a large apparent volume of distribution, and can significantly penetrate the blood-brain barrier.^9,10^ Abemaciclib is primarily metabolized in the liver by the cytochrome P450 enzyme CYP3A4, forming active metabolites M2 and M20.These metabolites may exhibit pharmacological activity and toxicity associated with the parent drug.^11,12^ However, detailed reports on their specific pharmacological activities and toxicological properties are currently unavailable. This metabolic pathway makes it susceptible to interactions with coadministered drugs, potent CYP3A4 inhibitors (e.g., clarithromycin) can significantly increase the plasma concentration of abemaciclib, while inducers (e.g., rifampin) can substantially reduce its exposure.^13^ It has been reported that letrozole exhibits mixed inhibition on the metabolism of abemaciclib *in vitro*, with particularly strong inhibitory effects against the CYP3A4.28 genotype.^14^ Additionally, both honokiol and astragaloside IV have been shown to inhibit CYP3A4 activity, leading to increased systemic exposure of abemaciclib in rats.^15,16^ Furthermore, when combined with endocrine drugs or other targeted therapies, potential metabolic interference may affect therapeutic efficacy or exacerbate adverse reactions (e.g., diarrhea, neutropenia), and even impact patients’ long-term quality of life.^17,18^ Therefore, clarifying the drug–drug interaction profile of abemaciclib is crucial for optimizing treatment regimens, managing toxicity, and improving patient tolerance.

This study aims to systematically evaluate the drug–drug interaction risk of abemaciclib through *in vitro* and *in vivo* experiments. We used a liver microsome incubation system to screen the effects of a series of drugs on abemaciclib metabolism, with a particular focus on its interaction with aromatase inhibitors like anastrozole. Molecular docking techniques were employed to elucidate the type of inhibition, which was subsequently validated in an SD rat model. The findings of this research will provide a pharmacokinetic basis for the precise clinical application of abemaciclib, aiding in the development of individualized dosing strategies and enhancing the safety and reliability of therapeutic efficacy assessments.

## 2 Materials and Methods

### 2.1 Chemicals and Reagents

Abemaciclib (purity >99%) and anastrozole (purity >99%) were supplied by Aladdin Reagent Co., Ltd. Ammonium acetate (purity >98%) was provided by Shanghai Macklin Biochemical Technology Co., Ltd. N-desethyl abemaciclib (M2) (purity >98%) was sourced from CHEMEGEN (Shanghai) Biotechnology Co., Ltd. Information on other drugs not detailed here is provided in the supplementary file. Nicotinamide adenine dinucleotide phosphate (NADPH) and hydroxy abemaciclib (M20) (purity >99%) were acquired from TargetMol, USA. Pooled female human liver microsomes (HLM) were purchased from Corning Life Sciences Co., Ltd. Female rat liver microsomes (RLM) were prepared in-house and their activity was validated following previously established protocols.^19^ Acetonitrile (HPLC grade), formic acid (HPLC grade), and methanol (HPLC grade) were products of Merck. All other chemicals used were of analytical grade.

### 2.2 Instrumentation and Analytical Conditions

Chromatographic separation was performed on a UPLC-MS/MS system fitted with a Waters UPLC BEH C18 column (2.1 mm × 50 mm, 1.7 μm particle size). The column temperature was maintained at 40 °C, while the autosampler temperature was set to 4 °C. The mobile phase consisted of solvent A (5 mM ammonium acetate containing 0.1% formic acid) and solvent B (acetonitrile), delivered at a flow rate of 0.5 mL/min with a total run time of 5 min. The gradient elution profile was as follows: 0–0.1 min, 90% A; 1.0–1.1 min, 90%–30% A; 1.1–4.0 min, 30% A; 4.0–4.1 min, 30%–90% A and 4.1–5.0 min, 90% A. Mass spectrometric detection was conducted using an AB Sciex Qtrap 6500+ triple quadrupole instrument operating in positive ionization mode with multiple reaction monitoring (MRM). The ion transitions monitored were *m*/*z* 507.00 **→**393.00 for abemaciclib, *m*/*z* 479.2 **→** 393.00 for M2, *m*/*z* 523.30 **→** 409.20 for M20, and *m*/*z* 237.10 **→** 194.10 for carbamazepine (internal standard, IS), respectively.

### 2.3 Enzyme Kinetic Studies

Incubation mixtures (200 μL total volume) comprised 100 mM phosphate-buffered saline (PBS, pH 7.4), 0.5 mg/mL female RLM or HLM, 1 mM NADPH, and varying concentrations of abemaciclib. Following a 5 min preincubation at 37 °C without NADPH, the enzymatic reaction was initiated by adding 1 mM NADPH. After 40 min of incubation, the reaction was quenched with 300 μL of ice-cold acetonitrile, followed by the addition of 20 μL IS (100 ng/mL carbamazepine). Samples were vortexed for 2 min and centrifuged at 13,000 rpm for 10 min, the resulting supernatant was collected for UPLC-MS/MS analysis.

For half-maximal inhibitory concentration (IC_50_) determinations, anastrozole was tested at concentrations of 0.01, 0.1, 1, 10, 25, 50, and 100 μM, while abemaciclib was held constant at 10 μM in female RLM and 30 μM in female HLM according to their respective K_m_ values. To elucidate the inhibition mechanism, abemaciclib concentrations in female RLM were set at 2.5, 5, 10, and 20 μM based on its K_m_, and anastrozole was tested at 12.5, 25, 37.5, and 50 μM derived from its IC_50_. In female HLM, abemaciclib was evaluated at 7.5, 15, 30, and 60 μM, with anastrozole at concentrations 0, 32.5, 65, and 97.5 μM. All subsequent procedures were identical to those described above.

Additional experiments were conducted using recombinant CYP3A4 isoforms. A 200 μL incubation system contained 100 mM PBS (pH 7.4), 7.5 μg CYP3A4.1 or CYP3A4.28, 10 μg cytochrome B5, 1 mM NADPH, and 6 μM abemaciclib (selected based on its K_m_). Anastrozole was added at concentrations of 0.01, 0.1, 1, 10, 25, 50, and 100 μM, and sample processing followed the same protocol as previous experiments.

### 2.4 Animal Studies

Female Sprague–Dawley rats (231.1 ± 13.8 g) were sourced from Zhejiang Vital River Experimental Animal Technology Co.; Ltd. Animals were fasted overnight but had free access to water. Ten rats were randomly divided into two groups (A and B): Group B received anastrozole (0.105 mg/kg/d) orally for 1 week prior to the experiment. After 1 week, both groups received a single oral dose of abemaciclib (15.75 mg/kg). Drug doses were calculated as bioequivalent based on clinical regimens. All compounds were dissolved in 1% CMC-Na. On the seventh day, anastrozole was administered orally, followed by abemaciclib 30 min later. Rats were allowed access to food for 4 h postdosing. Blood plasma samples were collected from the tail vein at 1, 2, 3, 4, 6, 8, 12, 24, 48, and 72 h. For analysis, 50 μL plasma was mixed with 150 μL acetonitrile and 20 μL of IS (100 ng/mL carbamazepine). The mixture was vortexed for 2 min and centrifuged at 13,000 rpm for 10 min, and the supernatant was subjected to UPLC-MS/MS analysis.

### 2.5 Western Blot Analysis

Liver tissues from female rats were homogenized in RIPA lysis buffer at 4 °C and centrifuged at 10,000 rpm. The supernatants were collected for protein analysis. Protein concentrations were measured using a BCA kit (Beyotime). Equal amounts of protein (30 μg) were separated by SDS-PAGE and transferred onto PVDF membranes. Membranes were blocked with 5% nonfat milk for 2 h, then incubated overnight with primary antibodies against CYP3A4 (Abcam ab3572, 1:1000 dilution) and GAPDH (Proteintech, 81640–5-RR, 1:10,000 dilution). After washing, membranes were incubated with HRP-conjugated antirabbit secondary antibody (1:10,000 dilution) for 2 h at room temperature. Protein bands were visualized using a chemiluminescence imaging system, and band intensities were quantified with ImageJ software.

### 2.6 Molecular Docking Analysis

Three-dimensional structures of abemaciclib and anastrozole were retrieved from the PubChem database in SDF format. The protein structure of CYP3A4 (Uniprot ID: P08684) was obtained from the UniProt database. Ligands and water molecules were removed from the protein structure, and hydrogen atoms were added using AutoDockTools. Molecular docking simulations and binding energy calculations were performed using AutoDock Vina. The resulting docking poses were visualized and analyzed with PyMOL software.

### 2.7 Statistical Analysis

Kinetic parameters (Michaelis–Menten, IC_50_) and plasma concentration–time profiles were generated using GraphPad Prism 9.0. Pharmacokinetic parameters were determined by noncompartmental analysis using MAS software. Statistical comparisons between two groups were conducted using Student’s *t* test in SPSS (version 26.0; SPSS Inc., Chicago, IL, USA). A *P*-value <0.05 was considered statistically significant.

## 3 Result

### 3.1 Anastrozole Significantly Inhibited the *In Vitro* and *In Vivo* Metabolism of Abemaciclib

To investigate potential drug–drug interactions involving abemaciclib, we built upon a preliminary drug screening experiment which found that anastrozole inhibited the formation of the M2 metabolite by over 70%. Given that these two drugs are frequently coadministered in clinical practice, we first conducted an *in vitro* inhibition assay using female rat liver microsomes (female RLM). As shown in Figure 1A, the half-maximal inhibitory concentration (IC_50_) of anastrozole on the metabolism of abemaciclib to M2 was 26.763 ± 1.991 μM, whereas the IC_50_ for the other metabolite, M20, was 83.603 ± 7.515 μM, indicating a stronger inhibitory effect of anastrozole on the M2 formation pathway.

**Figure 1:**
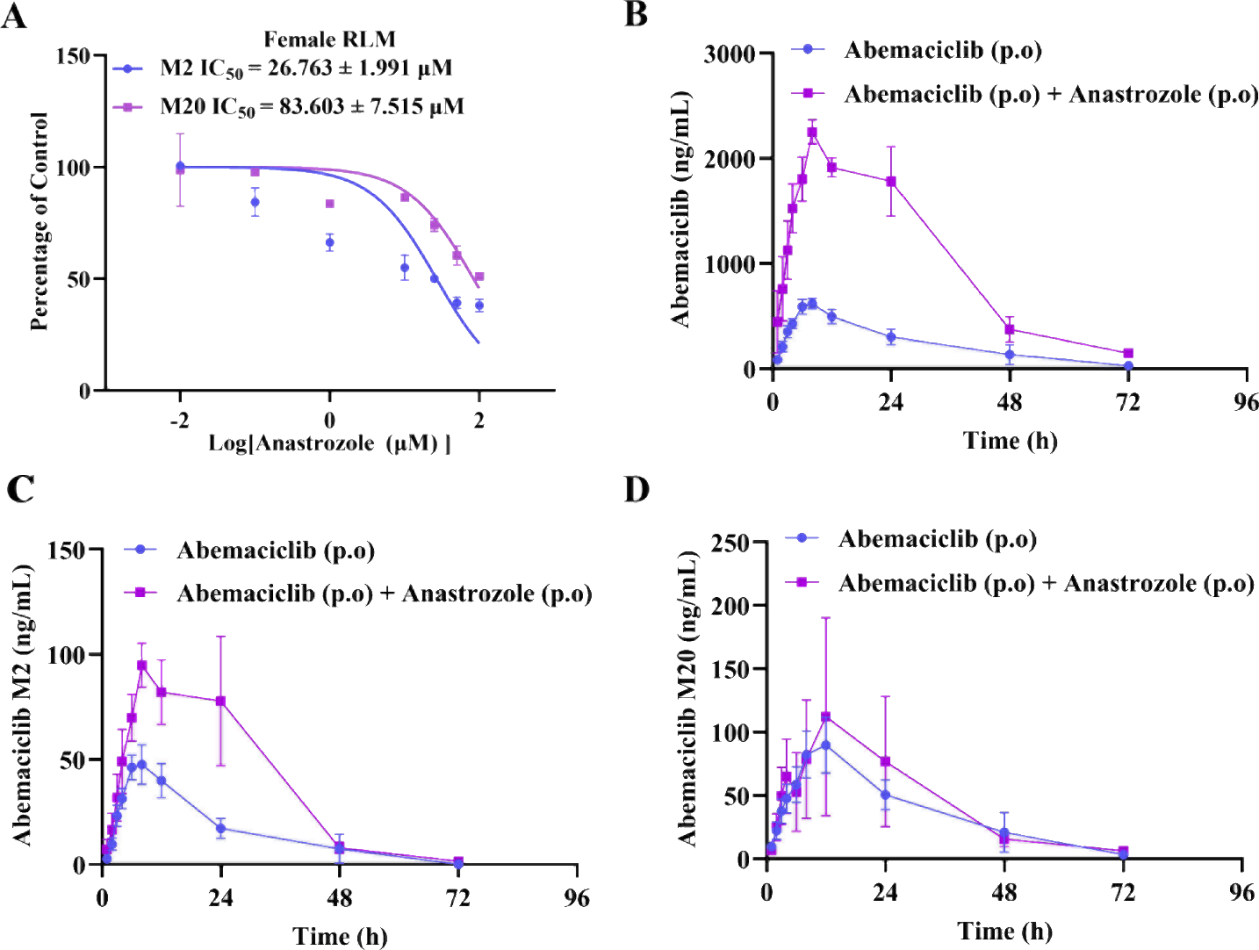
Anastrozole inhibits the metabolism of abemaciclib both *in vitro* and *in vivo*. (A) Inhibition curves of anastrozole on the *in vitro* metabolism of abemaciclib in female RLM. Data are presented as the mean ± SD, *n* = 3. (B-D) A graph was plotted with blood collection time as the *x*-axis and blood drug concentration of abemaciclib, M2 or M20 as the *y*-axis. The curves of abemaciclib when administered alone or in combination with anastrozole. Data are presented as the mean ± SEM, *n* = 5.

To further validate the *in vitro* findings, we conducted an *in vivo* study. Given that abemaciclib is primarily used in female patients, female SD rats were chosen as the animal model to reflect this clinical context. The animals were fasted before the experiment to eliminate dietary interference. As shown in Figure 1B and Table 1, the pharmacokinetic parameters of abemaciclib changed significantly after oral coadministration with anastrozole: the area under the concentration–time curve (AUC_0–t_ and AUC_0–∞_) increased by 326.6% and 271.9%, respectively, and the peak concentration (*C*_max_) increased by 273.3%. Additionally, the apparent volume of distribution (V_z/F_) decreased by 71.4%, and the oral clearance (CL_z/F_) decreased by 76.1%. Concurrently, as depicted in Figure 1C,D, the *C*_max_ of the primary metabolite M2 also showed an upward trend, further supporting the inhibitory effect of anastrozole on abemaciclib metabolism.

**Table 1:** Main Pharmacokinetic Parameters of Abemaciclib in Three Groups of Female SD Rats^a^

	Parameters	Abemaciclib (p.o)	Abemaciclib (p.o) + Anastrozole (p.o)
**Abemaciclib**	AUC_(0‑t)_ (μg/L·h)	16,084.02 ± 9,172.35	68,615.13 ± 17,189.44***
	AUC_(0‑∞)_ (μg/L·h)	18,370.38 ± 9,819.27	68,320.43 ± 17,636.65**
	*t*_1/2z_ (h)	10.06 ± 4.14	11.07 ± 3.83
	*T*_max_ (h)	7.20 ± 1.10	10.80 ± 7.43
	V_z/F_(L/kg)	12.92 ± 1.41	3.69 ± 0.99***
	CL_z/F_(L/h/kg)	1.010 ± 0.40	0.24 ± 0.06*
	*C*_max_ (ng/mL)	647.26 ± 126.90	2,416.22 ± 306.03***
			
**M2**	AUC_(0‑t)_ (μg/L·h)	947.60 ± 535.27	2,818.68 ± 1,617.57
	AUC_(0‑∞)_ (μg/L·h)	1,068.60 ± 791.85	1,680.49 ± 268.58
	*t*_1/2z_ (h)	5.03 ± 0.42	10.70 ± 4.25
	*T*_max_ (h)	8.00 ± 2.19	14.00 ± 9.17
	V_z/F_(L/kg)	176.92 ± 151.00	154.034 ± 88.62
	CL_z/F_(L/h/kg)	24.99 ± 22.83	9.55 ± 1.63
	*C*_max_ (ng/mL)	52.32 ± 15.58	111.62 ± 40.37**
			
**M20**	AUC_(0‑t)_ (μg/L·h)	2,666.53 ± 1,949.58	3,455.27 ± 3,107.44
	AUC_(0‑∞)_ (μg/L·h)	3,226.40 ± 1,818.10	3,234.98 ± 3,691.71
	*t*_1/2z_ (h)	9.79 ± 3.83	10.14 ± 1.17
	*T*_max_ (h)	11.33 ± 1.63	8.80 ± 3.35
	V_z/F_(L/kg)	75.59 ± 25.26	157.02 ± 112.86
	CL_z/F_(L/h/kg)	5.79 ± 2.08	10.22 ± 7.03
	*C*_max_ (ng/mL)	90.99 ± 53.75	104.96 ± 105.83

a **Notes:** AUC, area under the blood concentration–time curve; *t*_1/2z_, elimination half time; *T*_max_, peak time; V_z/F_, apparent volume of distribution; CL_z/F_, blood clearance; *C*_max_, maximum blood concentration. *P < 0.05, **P < 0.01, ***P < 0.001. compared with the Abemaciclib (p.o) group.

### 3.2 Mixed-Type Inhibition of Abemaciclib Metabolism by Anastrozole *In Vitro*

To elucidate the mechanism of inhibition by anastrozole, this study employed the IC_50_ shift assay method. As illustrated in Figure 2A,B, the IC_50_ shift results demonstrated that anastrozole exhibited nontime-dependent inhibition of abemaciclib metabolism in female rat liver microsomes (female RLM). The Western blot results in Figure 2C further indicated that anastrozole treatment did not affect the protein expression level of CYP3A4 in the rat liver. According to the nonlinear fitting curves in Figure 2D and Table 2, in the female RLM system, the K_m_ value of abemaciclib increased with rising inhibitor concentrations, while *V*_max_ remained stable, a kinetic characteristic consistent with a competitive inhibition model.

**Table 2:** Inhibitory Mechanism of Anastrozole on Abemaciclib Metabolism^a^

	K_m_ (μM)	*V*_max_ (pmol/min/μg protein)
**female RLM**		
0.5 IC_50_	11.617 ± 1.953	0.058 ± 0.005
IC_50_	16.037 ± 3.129	0.054 ± 0.006
1.5 IC_50_	16.253 ± 3.550	0.056 ± 0.011
2 IC_50_	19.017 ± 2.803*	0.056 ± 0.002
**female HLM**		
0	14.727 ± 1.130	0.774 ± 0.010
0.5 IC_50_	15.483 ± 3.229	0.722 ± 0.049
IC_50_	16.467 ± 3.654	0.694 ± 0.063
1.5 IC_50_	21.177 ± 1.232*	0.687 ± 0.034

a **Notes:** Compared with 0 group. *P < 0.05. **P < 0.01. ***P < 0.001.

**Figure 2:**
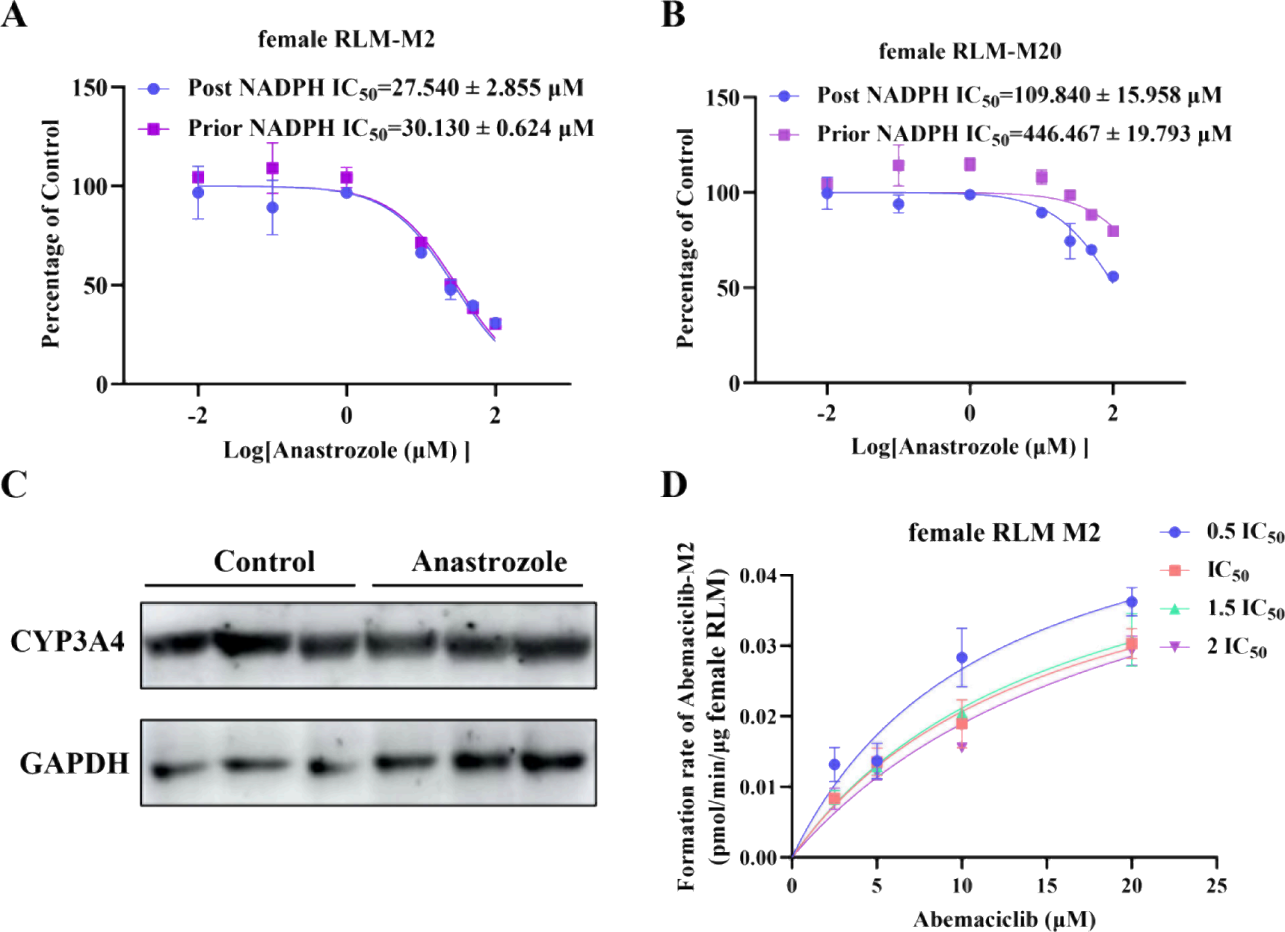
Mechanism of anastrozole’s inhibition of abemaciclib metabolism in female RLM. (A,B) NADPH was added before to or after incubation in the results of female RLM IC_50_ shift experiments. Data are presented as the mean ± SD, *n* = 3. (C) Western blot of CYP3A4 expression in liver after abemaciclib administration alone or in combination with anastrozole. (D) The nonlinear fitting plots showed the effects of different concentrations of anastrozole on the Michaelis–Menten curve of abemaciclib in female RLM. Data are presented as the mean ± SD, *n* = 3.

To further validate this inhibitory effect in a human-relevant model, we assessed the metabolic inhibition of abemaciclib in female human liver microsomes (female HLM). As per the IC_50_ curves in Figure 3A,B, the IC_50_ values for anastrozole against the formation of metabolites M2 and M20 in female HLM were 67.660 ± 3.151 μM and 236.867 ± 20.604 μM, respectively. The IC_50_ shift results in Figure 3C,D indicated that the inhibitory effect of anastrozole on abemaciclib was also nontime-dependent in female HLM. Through nonlinear fitting analysis shown in Figure 3E and Table 2, it was found that as the concentration of anastrozole increased, the K_m_ value of abemaciclib gradually rose while the *V*_max_ tended to decrease, a kinetic behavior suggesting a mixed model of competitive and noncompetitive inhibition.

**Figure 3:**
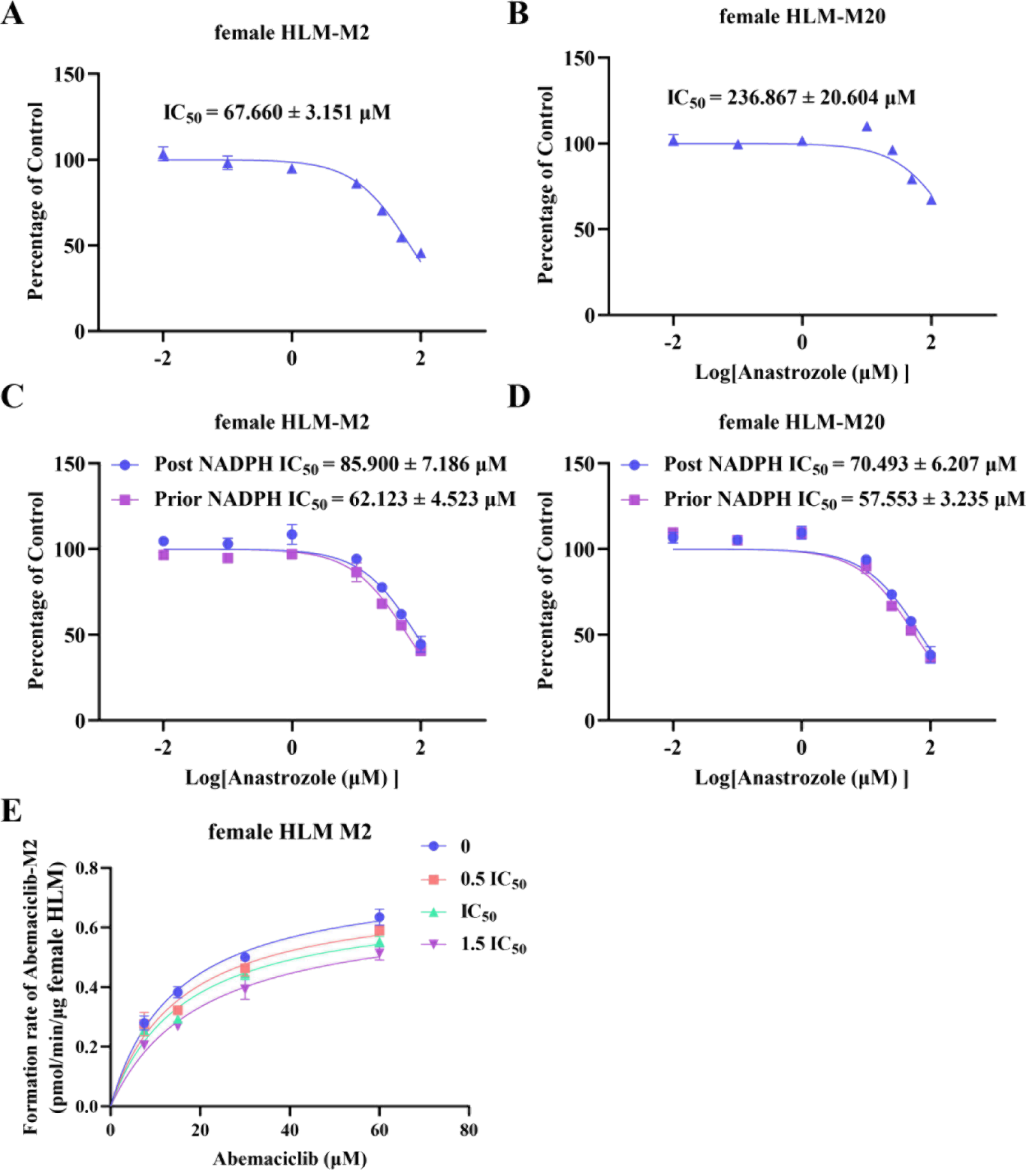
Characteristics of anastrozole’s inhibition of abemaciclib metabolism in female HLM. (A,B) Inhibition curves of anastrozole on the *in vitro* metabolism of abemaciclib in female HLM. (C,D) NADPH was added before to or after incubation in the results of female HLM IC_50_ shift experiments. (E) The nonlinear fitting plots showed the effects of different concentrations of anastrozole on the Michaelis–Menten curve of abemaciclib in female HLM. (A–E) Data are presented as the mean ± SD, *n* = 3.

### 3.3 The Effect of Anastrozole on Abemaciclib Metabolism in Different CYP3A4 Variants

Given that CYP3A4 is the primary enzyme mediating abemaciclib metabolism and based on our prior research on the impact of CYP3A4 genetic polymorphisms on abemaciclib metabolism, we investigated the inhibitory potency of anastrozole across different CYP3A4 variants. The results, as shown in Figure 4A,B, revealed that the IC_50_ values for anastrozole in CYP3A4.1 were 92.517 ± 11.491 μM (for M2) and 58.423 ± 9.053 μM (for M20). In the more active variant, CYP3A4.28, the IC_50_ values were 39.263 ± 1.136 μM (for M2) and 64.607 ± 5.130 μM (for M20). In contrast, when incubated with the low-activity variant CYP3A4.30, the levels of M2 and M20 generated were too low to reliably determine IC_50_ values.

**Figure 4:**
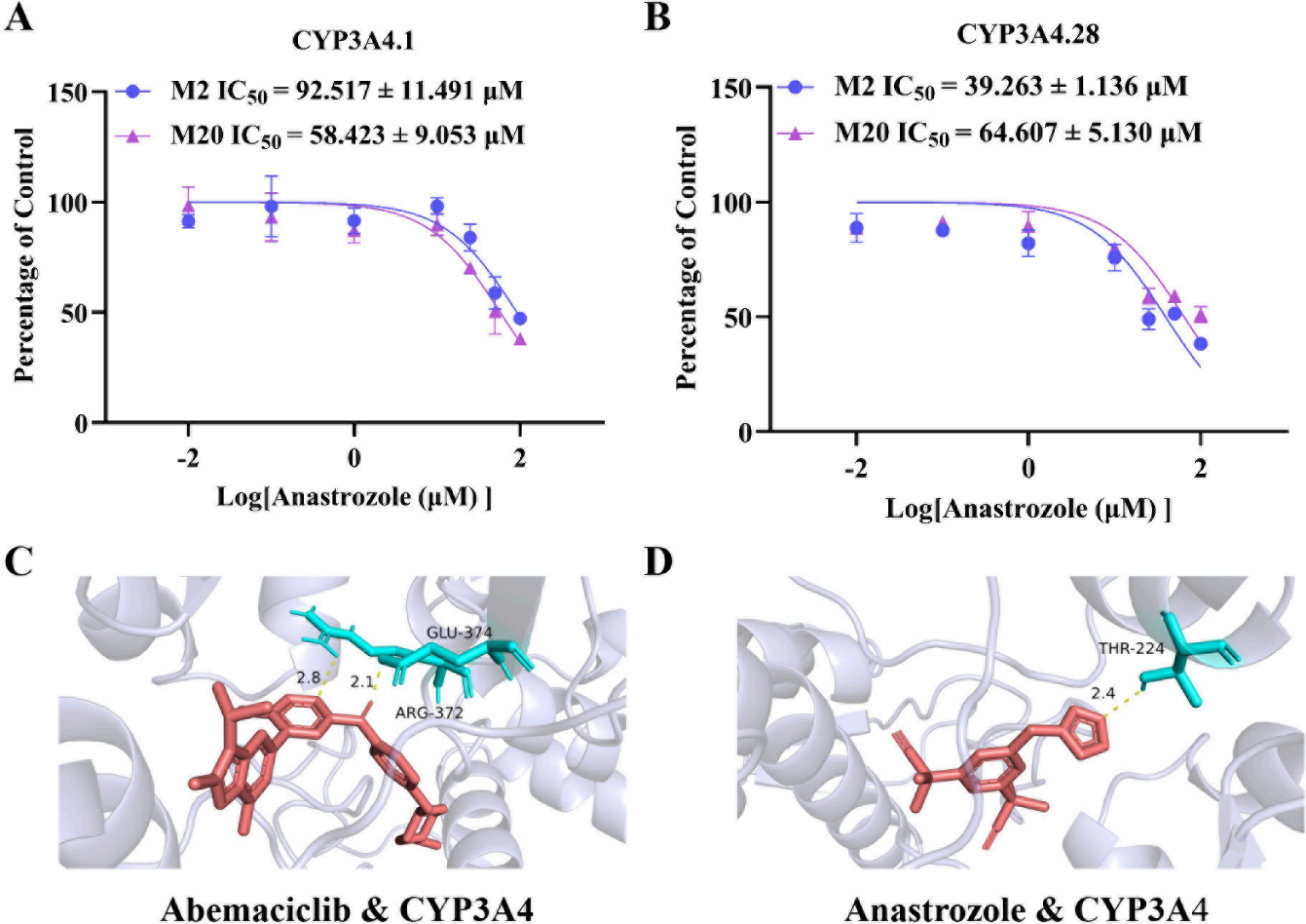
Effect of CYP3A4 on abemaciclib metabolism. (A,B) Inhibition curves of anastrozole on the *in vitro* metabolism of abemaciclib in CYP3A4.1/CYP3A4.28 baculosomes. Data are presented as the mean ± SD, *n* = 3. (C,D) Molecular docking results of CYP3A4 crystal structure with abemaciclib/anastrozole.

To elucidate the structural basis of this inhibitory action, this study employed molecular docking to examine the binding characteristics of abemaciclib and anastrozole with CYP3A4. As depicted in Figure 4C,D, the docking scores abemaciclib and anastrozole with CYP3A4 were −9.6 kcal/mol and −9.1 kcal/mol, respectively.

## 4 Discussion

Abemaciclib, a highly selective oral inhibitor of CDK4/6, effectively induces G1 phase cell cycle arrest by specifically blocking the CDK4/6-retinoblastoma protein signaling pathway, thereby suppressing tumor cell proliferation.^20^ The drug is now widely used in the treatment of HR+/HER2- advanced or metastatic breast cancer. Its excellent central nervous system penetration and sustained target inhibition can significantly prolong patients’ progression-free survival. However, abemaciclib can also cause dose-dependent adverse reactions such as diarrhea and neutropenia. Notably, abemaciclib is primarily metabolized by CYP3A4 of the cytochrome P450 enzyme system. Consequently, when coadministered with CYP3A4 inhibitors or inducers, its plasma exposure levels can change significantly, which in turn may affect efficacy or increase the risk of toxicity.^8,21,22^ This highlights the importance of thoroughly investigating the drug–drug interactions (DDI) of abemaciclib for safe clinical use.

In clinical practice, abemaciclib is often combined with aromatase inhibitors, such as anastrozole, for patients with HR+/HER2- breast cancer.^23^ Some clinical studies have indicated that this combination does not significantly alter the pharmacokinetic profile of abemaciclib. However, there are also reports suggesting that this combination may trigger serious adverse events, such as lung injury or acute respiratory distress syndrome, and even lead to patient death, with a potential underlying mechanism related to increased drug exposure.^24,25^ Our previous *in vitro* screening experiments found that anastrozole can significantly inhibit the metabolism of abemaciclib.^14^ This study further explores the interaction mechanism between abemaciclib and anastrozole through systematic *in vitro* and *in vivo* experiments. Understanding their interaction at the molecular level is of significant clinical importance for optimizing combination therapy regimens, assessing exposure risks, and guiding individualized dose adjustments.

This study utilized female SD rats for *in vivo* experiments to better simulate the clinical scenario where abemaciclib is primarily used in female breast cancer patients. Doses were administered after being converted to an equivalent based on clinically recommended dosages, and pharmacokinetic evaluation was conducted after a 7-day pretreatment with anastrozole. The main reason for considering the 7-day administration is that anastrozole can achieve a steady-state plasma concentration *in vivo*. The results showed that coadministration with anastrozole led to a sharp increase in abemaciclib exposure: its AUC_(0–t)_ and AUC_(0–∞)_ increased by 326.6% and 271.9%, respectively, and its *C*_max_ rose by 273.3%. This change is directly related to the significant decreases in the V_z/F_ and CL_z/F_, which fell by 71.4% and 76.1%, respectively, indicating that anastrozole severely hinders the *in vivo* elimination of abemaciclib. It is noteworthy that after coadministration, the blood exposure levels of abemaciclib and its metabolites M2 and M20 all showed an upward trend. From a metabolic perspective, if the metabolism of abemaciclib were inhibited, the formation of its metabolites should decrease. However, CYP enzymes (like CYP3A4) are present not only in the liver but also in the small intestine; when CYP enzyme activity is inhibited, it may simultaneously reduce both intestinal metabolism and the hepatic first-pass effect, thereby increasing the bioavailability of the parent drug.^26–28^ The significant decrease in the CL_z/F_ value supports this hypothesis. Given narrow therapeutic window of abemaciclib, such a more than 3-fold increase in exposure could significantly heighten the risk of dose-dependent adverse reactions like diarrhea and neutropenia, which warrants high clinical attention.

In the investigation of the inhibition mechanism, we utilized liver microsomes from different species for evaluation. Anastrozole exhibited competitive inhibition in female RLM, with an increased K_m_ and unchanged *V*_max_, suggesting it directly competes with abemaciclib for the active site of the CYP3A4 enzyme. In female HLM, however, a mixed-type inhibition and noncompetitive inhibition was observed, indicating that anastrozole can bind not only to the active site but also potentially to an allosteric site to interfere with substrate metabolism. This species-specific difference may stem from inherent structural variations between human and rat CYP3A enzymes.^29^ Western blot analysis confirmed that anastrozole does not affect the protein expression level of CYP3A4, ruling out the possibility that it affects enzyme activity through regulation of its degradation or synthesis. Nevertheless, this study has not yet directly verified the specific mechanism underlying the increased bioavailability, which warrants further investigation.

CYP3A4 plays a critical role in drug metabolism and is highly polymorphic, leading to significant interindividual variability in its phenotypic activity.^30–32^ As the primary enzyme system for the oxidative metabolism of abemaciclib, genetic variations in CYP3A4 can directly influence abemaciclib exposure levels and clinical efficacy. Our previous research found that compared to the wild-type CYP3A4.1, the variant CYP3A4.28 exhibits higher metabolic activity, while the catalytic activity of CYP3A4.30 is significantly reduced.^14^ This study further assessed the inhibitory potency of anastrozole across different CYP3A4 variants and found that genetic polymorphisms can significantly affect its inhibitory strength. In the high-activity CYP3A4.28 variant, anastrozole showed the strongest inhibitory activity on the primary metabolic pathway (formation of M2), suggesting that patients with this genotype face a higher risk of abemaciclib exposure when these drugs are used concomitantly. Furthermore, CYP3A4.28 has been previously identified in the Chinese population.^33^ It is worth noting that the anastrozole concentration used in this *in vitro* study was substantially higher than its clinically relevant exposure levels, suggesting that the observed inhibitory effect may be even more pronounced *in vivo*. In summary, these findings underscore the importance of CYP3A4 genotyping in personalized medicine and provide a theoretical basis for dose adjustments when combining these drugs in patients with different metabolic phenotypes.

In summary, this study comprehensively evaluated the drug–drug interaction between abemaciclib and anastrozole through a system of *in vitro* and *in vivo* experiments, further elucidating the DDI characteristics of abemaciclib. The results indicate that anastrozole can significantly increase the systemic exposure of abemaciclib by inhibiting its CYP3A4-mediated metabolism, which may alter its pharmacodynamic profile and increase the risk of adverse reactions. Therefore, extreme caution should be exercised during their clinical coadministration. Furthermore, this study found that CYP3A4 genetic polymorphisms can influence the inhibitory potency of anastrozole, providing a basis for individualized therapy for specific patient populations (such as those with different CYP3A4 genotypes) and pointing to new research directions for optimizing combination therapy in breast cancer.

## 5 Conclusion

Anastrozole inhibits the metabolism of abemaciclib in a nontime-dependent, mixed-type manner, altering its pharmacokinetic profile both *in vitro* and *in vivo*. Given the extensive clinical use of endocrine therapy that combines combining abemaciclib and anastrozole, this study provides a basis for the rational use of abemaciclib, supports individualized therapy, and helps prevent adverse reactions.

## Data Availability

The data that support the findings of this study are available on request from the corresponding author upon reasonable request.
